# RF_phage virion: Classification of phage virion proteins with a random forest model

**DOI:** 10.3389/fgene.2022.1103783

**Published:** 2023-02-08

**Authors:** Yanqin Zhang, Zhiyuan Li

**Affiliations:** ^1^ School of Finance, Xuzhou University of Technology, Xuzhou, China; ^2^ School of Artificial Intelligence and Software College, Jiangsu Normal University Kewen College, Xuzhou, China

**Keywords:** phage virion proteins, classification, bioinformatics, machine learning, random forest

## Abstract

**Introduction:** Phages play essential roles in biological procession, and the virion proteins encoded by the phage genome constitute critical elements of the assembled phage particle.

**Methods:** This study uses machine learning methods to classify phage virion proteins. We proposed a novel approach, RF_phage virion, for the effective classification of the virion and non-virion proteins. The model uses four protein sequence coding methods as features, and the random forest algorithm was employed to solve the classification problem.

**Results:** The performance of the RF_phage virion model was analyzed by comparing the performance of this algorithm with that of classical machine learning methods. The proposed method achieved a specificity (Sp) of 93.37%%, sensitivity (Sn) of 90.30%, accuracy (Acc) of 91.84%, Matthews correlation coefficient (MCC) of .8371, and an F1 score of .9196.

## 1 Introduction

Phages integrate their DNA sequences with bacterial genomes following infection and play a role in maintaining the diversity of microorganisms (Shen et al., 2007; Xia et al., 2010; Wetie Ngounou et al., 2014; Zou et al., 2016). If the abundance of a particular type of bacteria increases rapidly in a bacterial population, the corresponding phage specifically infects and kills the rapidly proliferating bacteria. The entire bacterial population returns to equilibrium following this process. Phages also participate in the Earth’s material cycle and are essential to the human microbiome (Brohee and Van Helden, 2006; Shen et al., 2019; Zhang and Quan, 2020). There are approximately 10^14^ bacteria in each individual’s gut, while the number of bacteriophages is 10^15−16^, which is ten times higher than the number of bacteria. These findings indicate that phage proteins play several crucial roles in biological processes (Ngo et al., 1994; Godzik et al., 1995; Whisstock and Lesk, 2003; Wu et al., 2009; De Las Rivas and Fontanillo, 2010; Awais et al., 2019).

Phage proteins can be classified as virion and non-virion proteins. The virion proteins encoded by the phage genes are essential components of the assembled phage particle and include the capsid protein, envelope protein, and virion enzymes (Chatterjee et al., 2011; You et al., 2013; Peng et al., 2017). These virion proteins determine the specificity for recognizing host bacteria and play essential roles in the recombination of phage viruses, receptor recognition, bacterial attachment, and penetration. The non-virion proteins of phages are synthesized in the infected cells and are also encoded by the phage genome. However, the non-virion proteins cannot be packaged into mature phage particles. The non-virion proteins primarily include enzymes and regulatory proteins, which play important roles in the processes of gene replication, transcription, and gene expression in phages (Sato et al., 1994; Schwikowski et al., 2000; Wei et al., 2017).

Several computational methods have been reported for classifying the functions of phage genes and virion proteins over the past few decades. Li et al. proposed a novel tool named SynFPS for classifying closely related genomes in whole genome comparison studies (Coates and Hall, 2003). The method employs a support vector machine (SVM) classifier and uses gene-to-gene distances as a feature. Feng et al. proposed a naïve Bayes method for classifying phage virion proteins based on the composition of primary amino acids and dipeptides as coding schemes (Free et al., 2009). Ding et al. proposed a method for classifying virion proteins using an SVM-based approach (Kim and Subramaniam, 2006). In these models, the key features among g-gap dipeptide compositions were initially determined by analysis of variance. Yang et al. described an ensemble algorithm-based method for classifying organellar proteins, in which the amino acid composition, physicochemical properties, sequence distribution, and structural characteristics of the sequences were used as features (Zhang et al., 2012). Han et al. proposed a two-layer multi-class SVM model for classifying subcellular localizations (Vazquez et al., 2003). After the first layer of SVM classification is completed, each amino acid sequence is represented by a k-dimensional vector, and each element in the vector corresponds to a classification result of the classifier (Yang et al., 2020). The output of the first layer is used as the input for the next layer, and the second layer uses SVM to determine the final result. Jia et al. proposed a random forest algorithm-based method that used different features extracted from protein sequences (You et al., 2017). The method used a voting system for computing the final classification results, which depended on seven independent models. Bahri et al. proposed an ensemble method named Greedy-Boost based on the adaptive combination, which improves the accuracy of detection (Guo et al., 2008). Although the smoothing method improves the stability of the classification system, the method has a high computational cost. Zhang et al. proposed a method based on logistic models for classifying samples using the amino acid composition, transformation, and distribution features and pseudo-amino acid composition as features (Koike and Takagi, 2004). The final results were computed based on the results obtained from the classification models. Liu et al. used different weights for classifying the four SVMs used in their study (You et al., 2015a). The method determined the final classification by traversing and selecting appropriate parameters. These findings indicate that ensemble algorithms can improve the accuracy of the final classification.

**FIGURE 1:**
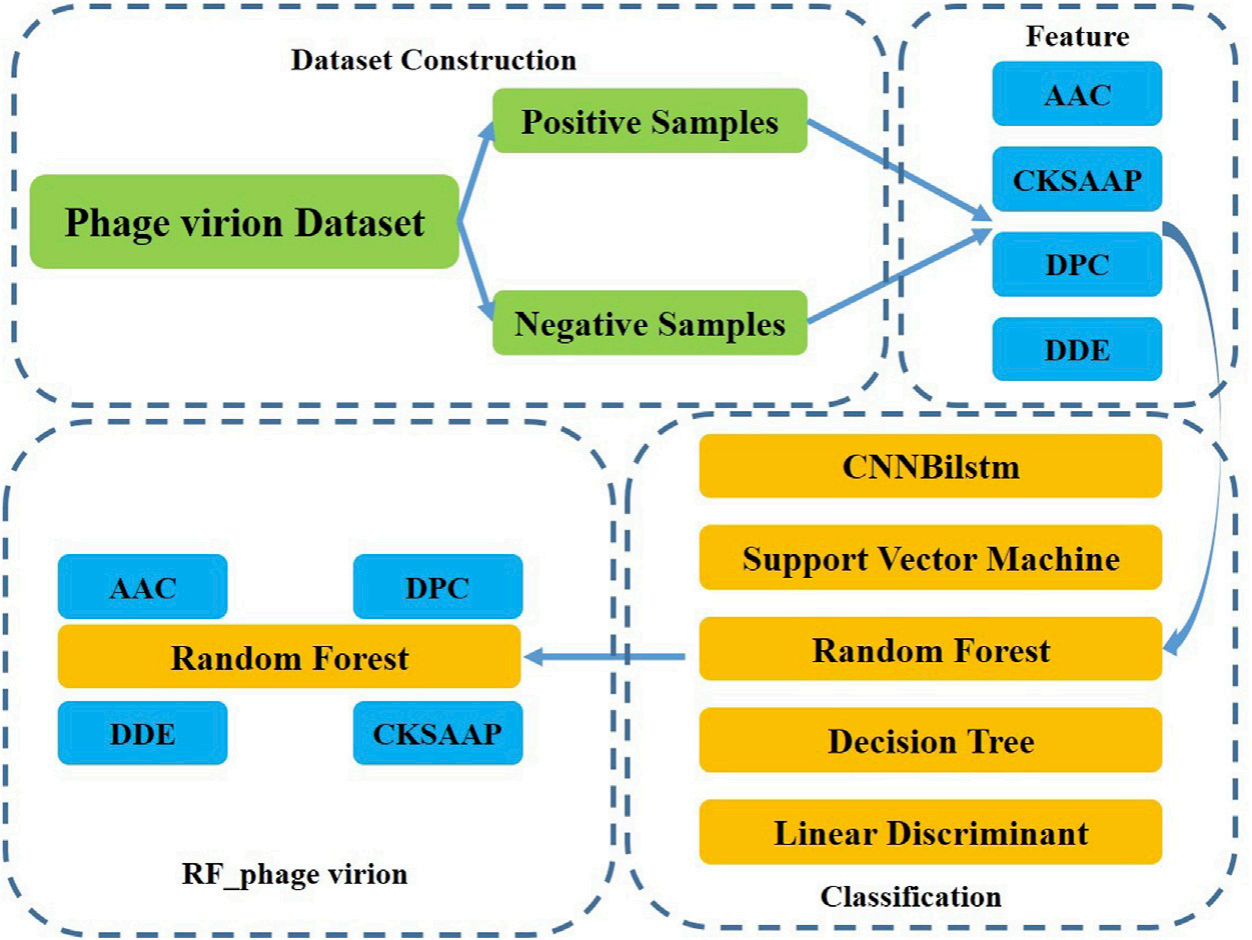
The outlines of RF_phage virion.

This study aimed to develop a method for the classification of phage virion proteins using machine learning methods. A novel method, RF_phage virion, is proposed herein for the effective classification of the virion and non-virion proteins. The method uses four protein sequence coding methods as features, and the random forest algorithm is used for solving the classification problem. The performance of the RF_phage virion model was determined by comparing the performance of this algorithm with some classical machine learning methods. A schematic representation of the RF_phage virion method is provided in Figure 1.

## 2 Materials and methods

### 2.1 Dataset

Ding’s dataset, which primarily focuses on phage virion proteins, was used for classifying the phage proteins in this study (Bradford and Westhead, 2005; Cui et al., 2012; Romero-Molina et al., 2019). Ding’s dataset comprises 1000 samples, of which phage virion proteins constitute one-half, and the other half comprises non-phage virion proteins. The dataset can be treated as a typical ideal dataset for the classification of phage virion proteins. There is a large difference between the number of non-phage and phage virion proteins. Therefore, Ding’s dataset can be considered an ideal benchmark dataset for phage virion protein classification problems. The detailed information of the employed dataset is demonstrated in Table 1.

**TABLE 1:** The information of dataset.

Non-phage virion proteins	Phage virion proteins
500	500

### 2.2 Encoding methods

#### 2.2.1 Amino acid composition (AAC)

The AAC feature describes the distribution of amino acid residues (Li et al., 2012). The feature focuses on the frequency of occurrence of each amino acid residue. At the same time, the AAC feature can provide typical statistical information regarding the identified protein sequences. The formula used for determining the AAC is provided in Eq. 1:

$$AAC=\frac{aac\left(i\right)}{length},i\in \left\{A,C,\cdots ,Y\right\}$$ (1)

Where, *length* represents the length of the identified phage virion protein sequence, and aac(*i*) represents the occurrence of the *i*th amino acid residue in the protein sequence. The parameter *i* refers to the twenty amino acids present in protein sequences. The sum of the twenty amino acids equals to 1.

#### 2.2.2 Composition of k-spaced amino acid pairs (CKSAAP)

Although the AAC feature includes the amino acids present in protein sequences, the feature does not provide any positional information regarding the amino acids in protein sequences (Chen and Liu, 2005; You et al., 2015b; Wang et al., 2018). The CKSAAP feature describes the relationship between two amino acid residues in protein sequences, and focuses on the frequency of amino acid residue pairs, which are separated by *n* number of neighboring amino acid residues. For instance, *n* = 0 indicates that the two amino acids are successive. There are 400 types of AACs, and CKSAAP can compute the frequency of occurrence for each combination. The formula used for determining the CKSAAP is provided in in Eq. 2:

$$CKSAAP\;\left(n=0\right)={\left(\frac{N_{AA}}{N_{total}},\frac{N_{AC}}{N_{total}},\cdots ,\frac{N_{AY}}{N_{total}},\cdots ,\frac{N_{YY}}{N_{total}}\right)}_{400}$$ (2)

In this study, the value of *n* was set to 3, and the scale of the CKSAAP feature can reach 1600.

#### 2.2.3 Di-peptide composition (DPC)

The DPC feature focuses on the correlation between two successive amino acid residues (Sun et al., 2017). The scale of this feature can reach 400. The DPC feature was calculated using the formula in Eq. 3:

$$DPC=\frac{bipeptide\left(i\right)}{length},i\in \left\{AA,AC,\cdots ,YY\right\}$$ (3)

Where, the sum of the whole elements equals 1. In other words, the DPC can be treated as a second-order term of amino acid pairs.

#### 2.2.4 Dipeptide deviation extraction (DDE)

The DDE feature focuses on a binomial and uniform distribution theoretical sequence, but does not consider the alignment of protein relationships (Zhang et al., 2019). The feature can elucidate the interrelationships within a set of proteins. There DDE feature comprises three key parameters, namely, the size of the dipeptide composition (Dc), the means of theoretical values (T_m_), and the theoretical value of variance (T_v_). The formula used for calculating the DDE is depicted in Eq. 4:

$$DDE\left(t\right)=\frac{D_{c}\left(i\right)-T_{m}\left(i\right)}{\sqrt{T_{V}\left(i\right)}}$$ (4)

For instance, two pairs of successive amino acid residues have a DPC of 400. The scale of the DDE feature is 400, as depicted in Eq. 5:

$$DDE\left(t=2\right)=\left\{{dde}_{i}\right\},i\in \left[0,400\right]$$ (5)

The formulae used for estimating the D_c_, T_m_, and T_v_ are provided in Eq. (6) (7) (8), provided hereafter.

$$D_{c}\left(i\right)=\frac{n_{i}}{N}$$ (6)

There are 400 combinations of amino acid pairs in each dipeptide. Therefore, the D_c_(*i*) can be treated as an element in related DPC features.

$$T_{M}\left(i\right)=\frac{C_{i1}}{C_{N}}\times \frac{C_{i2}}{C_{N}}$$ (7)

Where, T_m_ represents the theoretical average, C_i1_ represent the occurrence of the first amino acid residue, C_i2_ represents the occurrence of the second amino acid residue, and C_N_ represents the entire set of amino acids.

$$T_{v}\left(i\right)=\frac{T_{M}\left(i\right)\left(1-T_{M}\left(i\right)\right)}{N}$$ (8)

Where, T_v_ represents the theoretical variations in dipeptides.

### 2.3 Random forest algorithm

**FIGURE 2:**
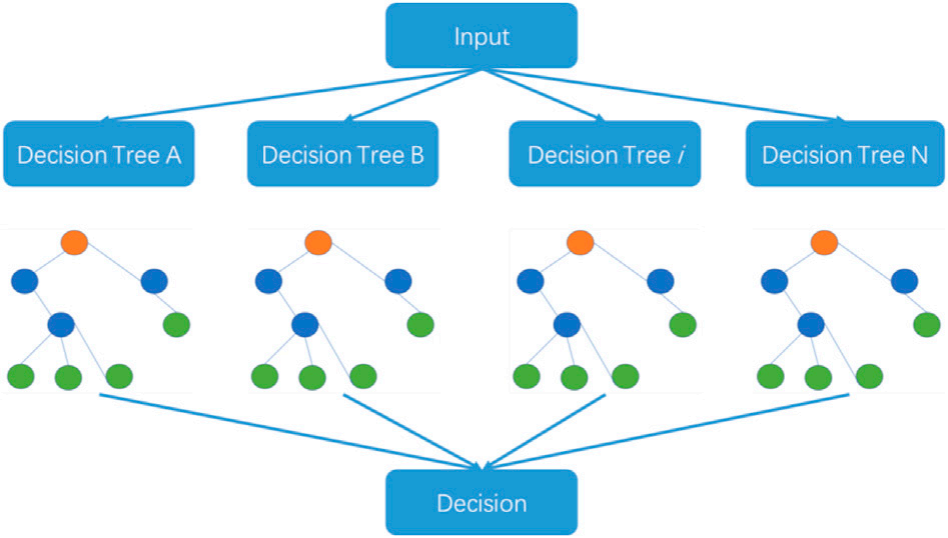
The structure of the random forest algorithm.

The random forest algorithm was proposed by L. Breiman at the beginning of this century and has been successfully used for dealing with classification and regression problems in related areas (Saha et al., 2014; Liu et al., 2018). The algorithm combines randomized decision trees and subsequently aggregates the average results from the decision trees. This algorithm can deal with high-dimensional small-sample problems. In other words, the algorithm performs well in identification problems using datasets where the scale of variables is much larger than the number of samples. The random forest algorithm is also used in big dataset problems. The steps of the random forest algorithm are outlined in Figure 2.

### 2.4 Measurement of performance

The samples in the classification problem in this study could be categorized into two, namely, phage and non-phage virion protein sequences. The defined positive samples comprised the virion protein sequences, while the defined negative samples comprised the non-phage protein sequences of phages. According to the definition, classified samples can produce four results under common conditions. These formulations, including the sensitivity (Sn), specificity (Sp), accuracy (ACC), F1 scores, and Matthews correlation coefficient (MCC), were obtained using the formulae in Eq. (4) (5) (6) (7) (8), provided hereafter.

$$Sn=\frac{TP}{TP+FN}$$ (4a)

$$Sp=\frac{TN}{TN+FP}$$ (5a)

$$Acc=\frac{TP+TN}{TP+TN+FP+FN}$$ (6a)

$$F1=\frac{2TP}{2TP+FN+FP}$$ (7a)

$$MCC=\frac{TP\times TN-FP\times FN}{\sqrt{\left(TP+FP\right)\left(TP+FN\right)\left(TN+FP\right)\left(TN+FN\right)}}$$ (8a)

Where, P and N represent the scale of positive and negative samples, respectively. T and F represent sets of true and false predicted results, respectively.

The F1 score is used to evaluate the distribution of positive and negative samples in two-types problems. Performance measures should consider several parameters, including the four basic parameters, namely, TP, FP, TN, and FN. The performance measure can be treated as a harmonic average of model accuracy and recall. Another important measure of performance is the MCC, and the values of this performance measure ranges from −1 to 1.

## 3 Results

The random forest model was used in this study for classifying the virion and non-virion proteins of phages using four typical protein features, namely, the AAC, CSKAAP, DPC, and DDE. The performance of the method was determined by comparing with state-of-the-art methods.

**FIGURE 3:**
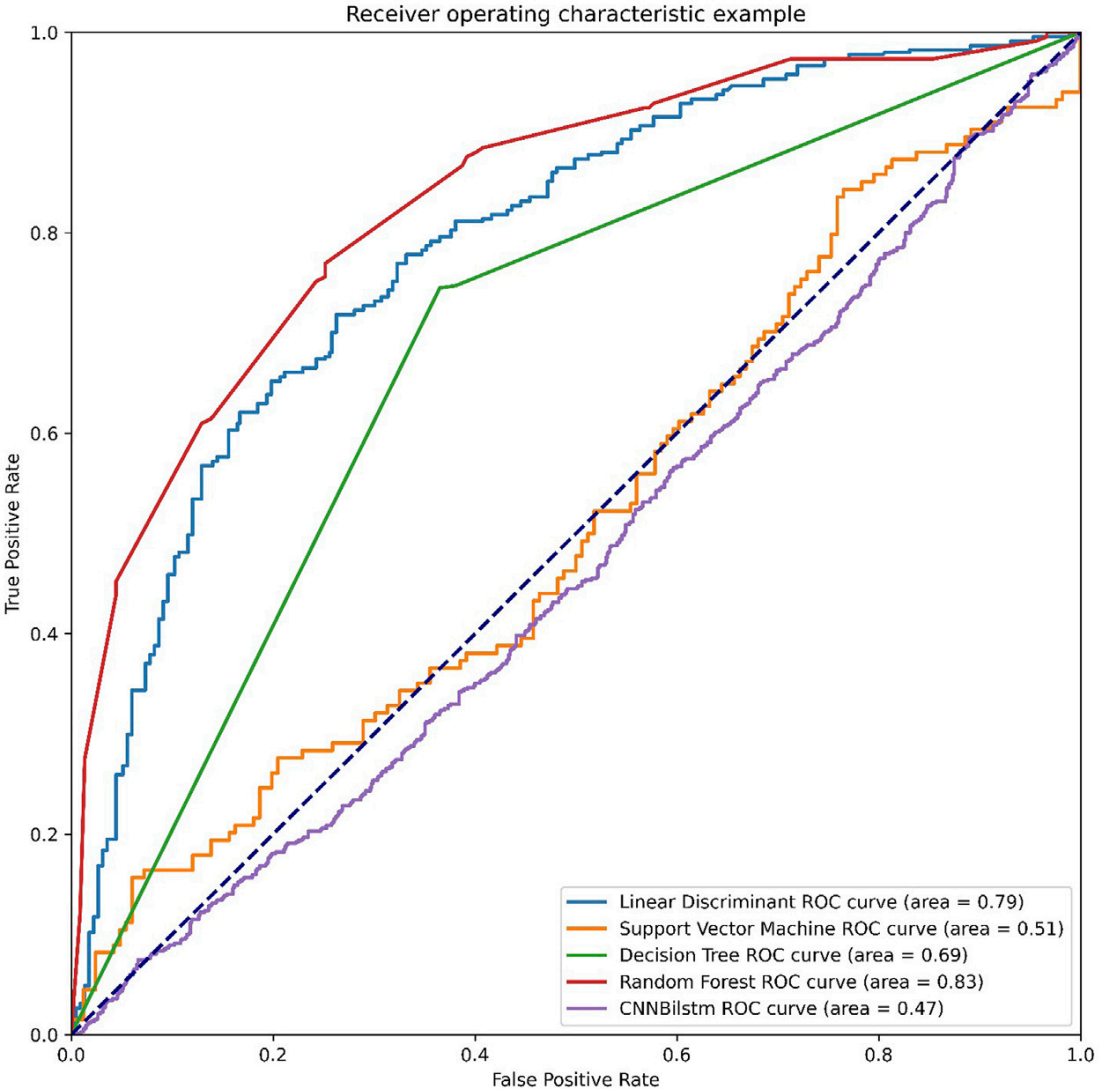
The ROC curves of AAC feature. Note: LD means the linear discriminant classifier. SVM means the support vector machine. DT means the decision tree. RF means the random forest and CNNBilstm mean convolution neural network with Bi-Long Short Term Memory.

As depicted in Figure 3 and Table 2, the values of Sp, Sn, Acc, MCC, and F1 score for the SVM-based method were 46.99%, 52.24%, 49.61%, −.0077, and .4825, respectively, while the values of these indices for the decision tree model were 63.47%, 74.50%, 68.99%, .3821, and .6718, respectively. The values of Sp, Sn, Acc, MCC, and F1 score for the random forest algorithm using the AAC feature were 74.83%, 76.94%, 75.89%, .5178, and .7563, respectively, while the values of these indices for the deep learning algorithm, which is a convolution neural network, were 99.47%, 0%, 49.74%, −.0514, and .6643, respectively.

**TABLE 2:** The performances of AAC feature.

	SP (%)	SN (%)	Acc (%)	MCC	F1 score
LD	74.16	70.07	72.12	4427	7268
SVM	46.99	52.24	49.61	−0077	4825
DT	63.47	74.50	68.99	3821	6718
RF	74.83	76.94	75.89	5178	7563
CNNBilstm	99.47	00	49.74	−0514	6643

**FIGURE 4:**
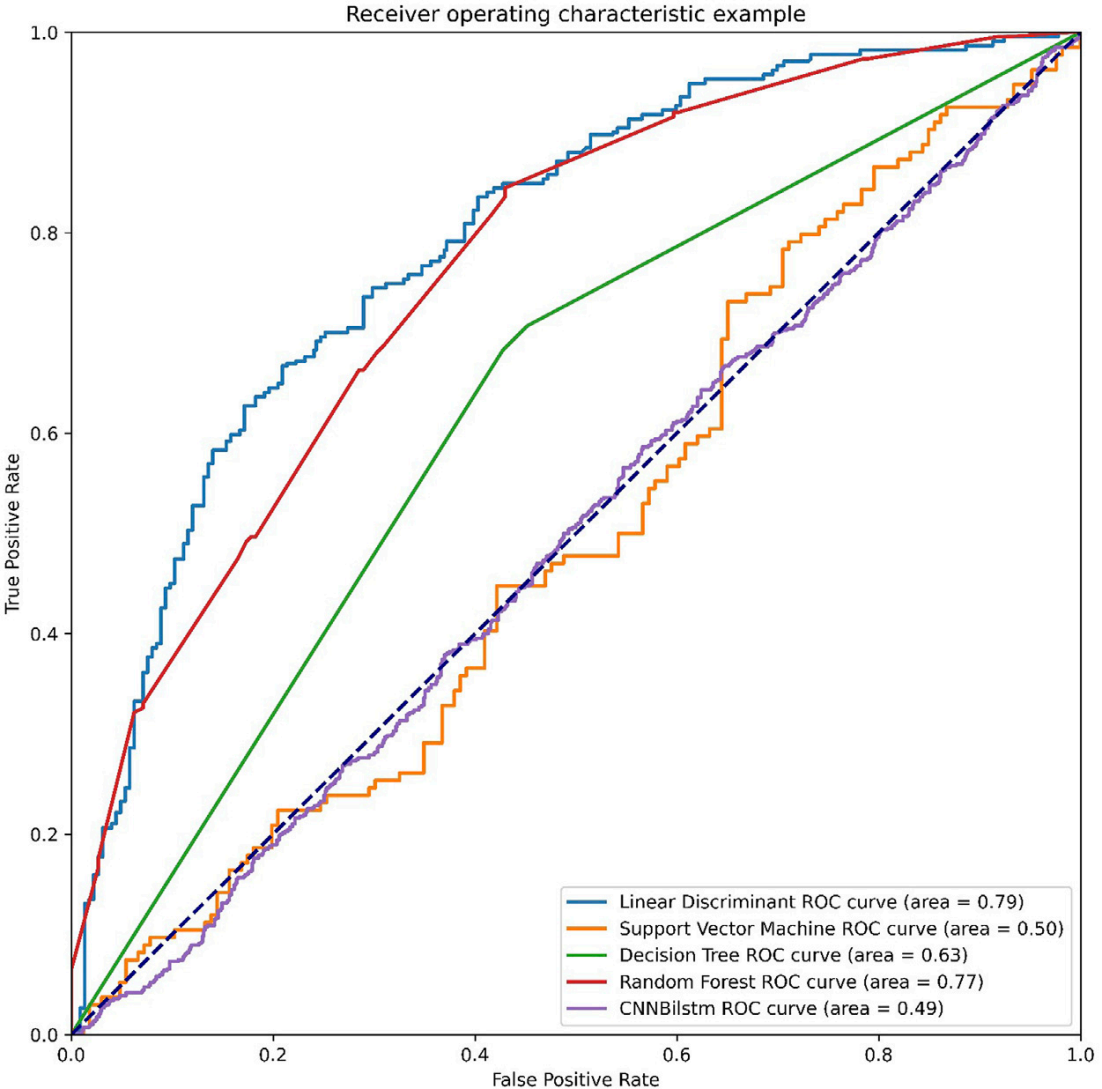
The ROC curves of CSKAAP feature.

As depicted in Figure 4 and Table 3, the values of Sp, Sn, Acc, MCC, and F1 score for the SVM-based method were 21.69%, 84.33%, 53.01%, .0772, and .3158, respectively, while the values of these indices for the decision tree model were 57.24%, 68.29%, 62.77%, .2569, and .6059, respectively. The values of Sp, Sn, Acc, MCC, and F1 score for the random forest algorithm using the CSKAAP feature were 69.04%, 68.74%, 68.89%, .3778, and .6894, respectively, while the values of these indices for the convolution neural network were 98.95%, 0%, 49.47%, −.0727, and .6620, respectively.

**TABLE 3:** The performances of CSKAAP feature.

	SP (%)	SN (%)	Acc (%)	MCC	F1 score
LD	77.73	67.18	72.46	4516	7384
SVM	21.69	84.33	53.01	0772	3158
DT	57.24	68.29	62.77	2569	6059
RF	69.04	68.74	68.89	3778	6894
CNNBilstm	98.95	00	49.47	−0727	6620

**FIGURE 5:**
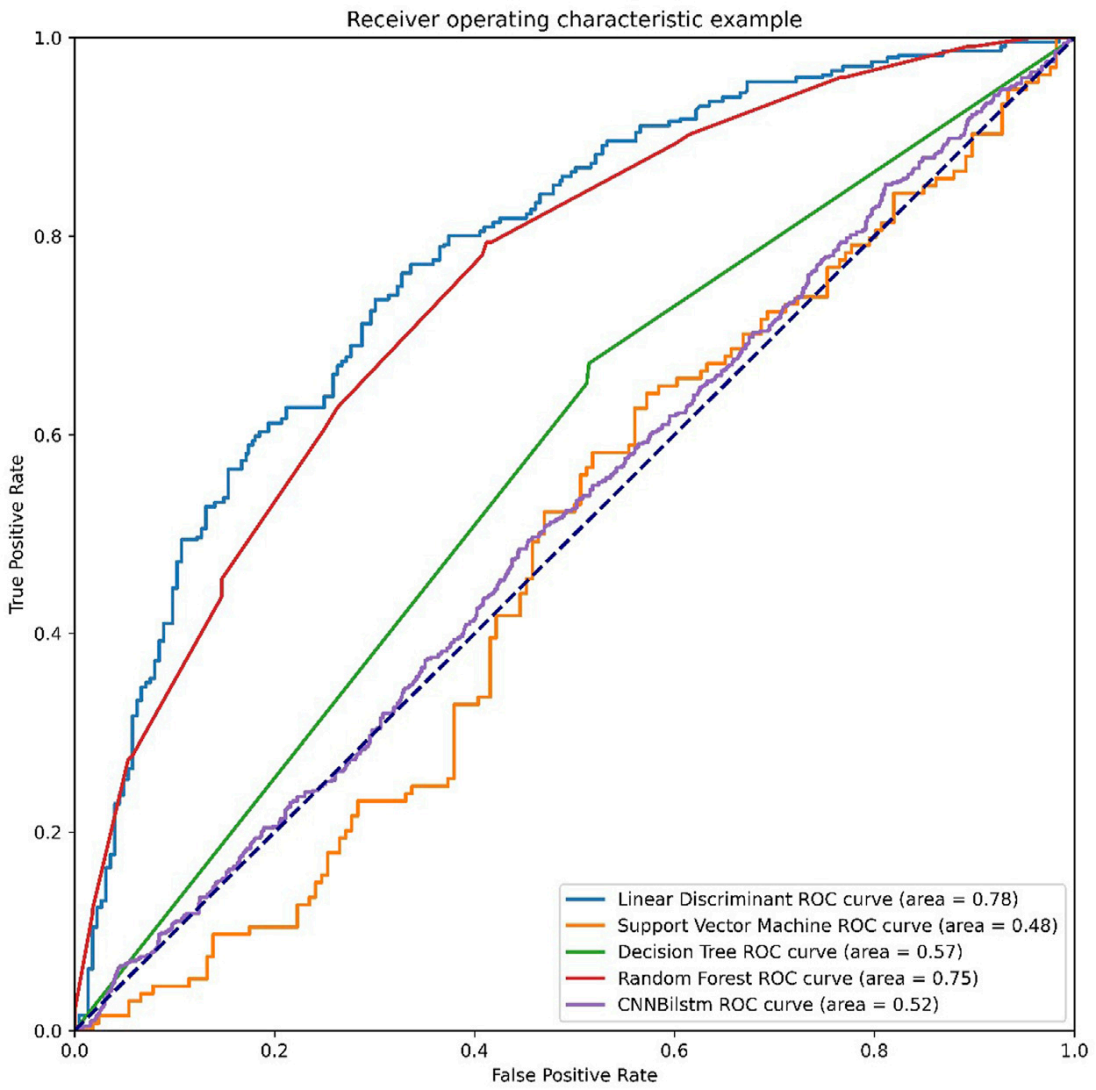
The ROC curves of DPC feature.

As depicted in Figure 5 and Table 4, the values of Sp, Sn, Acc, MCC, and F1 score for the SVM-based method were 54.82%, 75.37%, 65.10%, .3085, and .611, respectively, while the values of these indices for the decision tree model were 65.26%, 67.85%, 66.55%, .3312, and .6611, respectively. The values of Sp, Sn, Acc, MCC, and F1 score for the random forest algorithm using the DPC feature were 65.26%, 67.85%, 66.55%, .3312, and .6611, respectively, while the values of these induces for the convolution neural network were 88.44%, 0%, 44.22%, −.2477, and .6132, respectively.

**TABLE 4:** The performances of DPC feature.

	SP (%)	SN (%)	Acc (%)	MCC	F1 score
LD	97.33	13.08	55.20	1932	6848
SVM	36.75	67.16	51.96	0411	4334
DT	48.78	65.19	56.98	1416	5314
RF	73.72	62.75	68.23	3669	6989
CNNBilstm	95.90	00	47.95	−1446	6482

**FIGURE 6:**
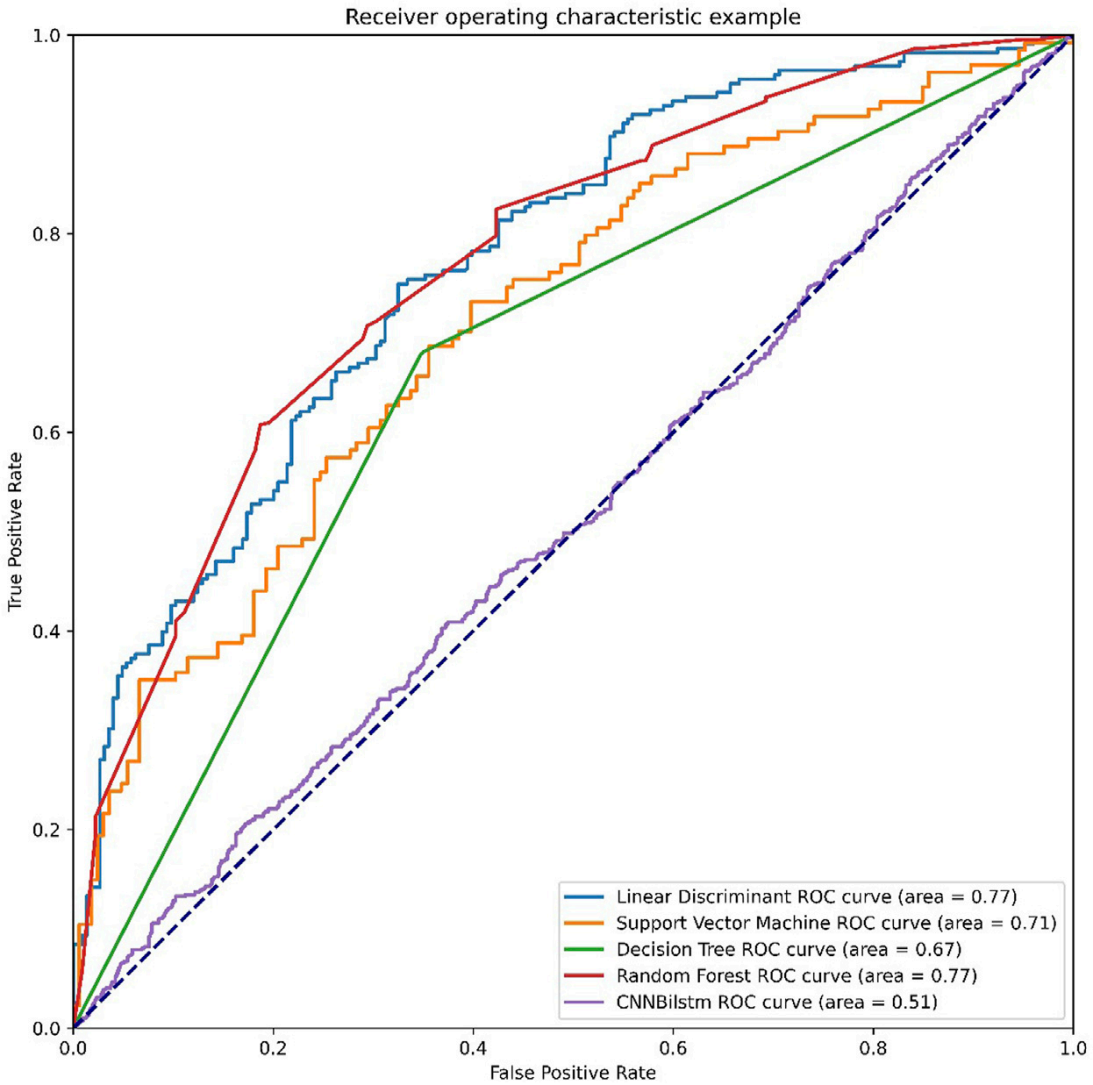
The ROC curves of DDE feature.

As depicted in Figure 6 and Table 5, the values of Sp, Sn, Acc, MCC, and F1 score for the SVM-based method were 36.75%, 67.16%, 51.96%, .0411, and .4334, respectively, while the values for the decision tree model were 48.78%, 65.19%, 56.98%, .1416, and .5314, respectively. The values of Sp, Sn, Acc, MCC, and F1 score for the random forest algorithm using the DDE feature were 73.72%, 62.75%, 68.23%, .3669, and .6989, respectively, while the values of these indices for the convolution neural network were 95.90%, 0%, 47.95%, −.1446, and .6482, respectively.

**TABLE 5:** The performances of DDE feature.

	SP (%)	SN (%)	Acc (%)	MCC	F1 score
LD	97.33	13.08	55.20	1932	6848
SVM	36.75	67.16	51.96	0411	4334
DT	48.78	65.19	56.98	1416	5314
RF	73.72	62.75	68.23	3669	6989
CNNBilstm	95.90	00	47.95	−1446	6482

## 4 Discussions

In the section of results, we merely employed the AAC, CSKAAP, DPC, and DDE features, respectively. Therefore, we combined the four features to evaluate the performances in this work.

**FIGURE 7:**
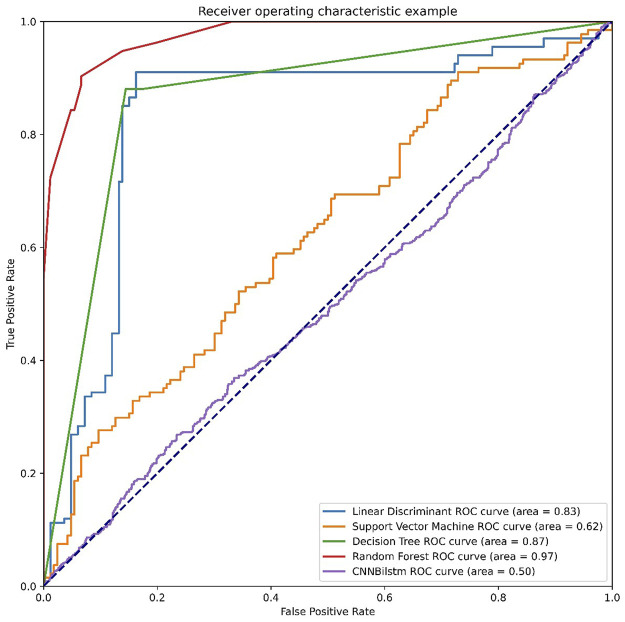
The ROC curves of combination feature.

As depicted in Figure 7 and Table 6, the values of Sp, Sn, Acc, MCC, and F1 score for the SVM-based method were 18.07%, 91.79%, 54.93%, .1460, and .2862, respectively, while the values for the decision tree model were 83.73%, 88.06%, 85.90%, .7186, and .8559, respectively. The values of Sp, Sn, Acc, MCC, and F1 score for the random forest algorithm using the combination feature were 93.37%, 90.30%, 91.84%, .8371, and .9196, respectively, while the values of these indices for the convolution neural network were 98.45%, .00%, 49.22%, −.0885, and .6597, respectively.

**TABLE 6:** The performances of combination feature.

	SP (%)	SN (%)	Acc (%)	MCC	F1 score
LD	84.34	86.57	85.45	7092	8529
SVM	18.07	91.79	54.93	1460	2862
DT	83.73	88.06	85.90	7186	8559
RF	93.37	90.30	91.84	8371	9196
CNNBilstm	98.45	00	49.22	−0885	6597

## 5 Conclusion

The present study uses machine learning methods to classify phage virion proteins. Four protein sequence coding methods, namely AAC, CSKAAP, DPC, and DDE, were used as features for the effective classification of the virion and non-virion proteins. The random forest algorithm was subsequently used to solve the classification problem. By combining each of the four features with the classification algorithm, we observed that the performance of the model was best when the combination feature was used.

When it comes to the problem of classification of phage virion proteins, such an issue can be regarded as a typical binary classification problem in the field of machine learning. In this work, we employed Ding’s dataset, which is a balanced dataset. Actually, the size of positive samples can hardly be equal to the size of the negative ones. In this work, the AAC, CSKAAP, DPC, and DDE feature and their combination feature can be employed as the input of the RF_phage virion model. There are several other features in the field of protein research. Therefore, these features can also be employed in future work. On the other hand, the other typical classification algorithm can be utilized in future work. The size of the combination feature can reach 2420. Considering such a situation, some reduced useless information approaches can be utilized in this future work.

## Data Availability

The original contributions presented in the study are included in the article/supplementary material, further inquiries can be directed to the corresponding author.
