# Corrigendum: RF_phage virion: classification of phage virion proteins with a random forest model

**DOI:** 10.3389/fgene.2023.1224665

**Published:** 2023-06-14

**Authors:** Yanqin Zhang, Zhiyuan Li

**Affiliations:** ^1^ School of Finance, Xuzhou University of Technology, Xuzhou, China; ^2^ School of Artificial Intelligence and Software College, Jiangsu Normal University Kewen College, Xuzhou, China

**Keywords:** phage virion proteins, classification, bioinformatics, machine learning, random forest

In the published article, an **Author name** was incorrectly written as “Yanqing Zhang.” The correct spelling is “Yanqin Zhang.”

The authors apologize for this error and state that this does not change the scientific conclusions of the article in any way. The original article has been updated.

